# Ca^2+^-Clock-Dependent Pacemaking in the Sinus Node Is Impaired in Mice with a Cardiac Specific Reduction in SERCA2 Abundance

**DOI:** 10.3389/fphys.2016.00197

**Published:** 2016-06-02

**Authors:** Sunil Jit R. J. Logantha, Mathis K. Stokke, Andrew J. Atkinson, Sanjay R. Kharche, Sajida Parveen, Yawer Saeed, Ivar Sjaastad, Ole M. Sejersted, Halina Dobrzynski

**Affiliations:** ^1^Institute of Cardiovascular Sciences, University of ManchesterManchester, UK; ^2^Institute for Experimental Medical Research, Oslo University Hospital and University of OsloOslo, Norway; ^3^Center for Heart Failure Research, University of OsloOslo, Norway; ^4^Clinic for Internal Medicine, Lovisenberg Deaconess Hospital ASOslo, Norway

**Keywords:** sinus node, atrioventricular node, Ca^2+^-clock, SERCA2, heart failure, telemetry, heart rate, mathematical model

## Abstract

**Background:** The sarcoplasmic reticulum Ca^2+^-ATPase (SERCA2) pump is an important component of the Ca^2+^-clock pacemaker mechanism that provides robustness and flexibility to sinus node pacemaking. We have developed transgenic mice with reduced cardiac SERCA2 abundance (*Serca2* KO) as a model for investigating SERCA2's role in sinus node pacemaking.

**Methods and Results:** In *Serca2* KO mice, ventricular SERCA2a protein content measured by Western blotting was 75% (*P* < 0.05) lower than that in control mice (*Serca2* FF) tissue. Immunofluorescent labeling of SERCA2a in ventricular, atrial, sinus node periphery and center tissue sections revealed 46, 45, 55, and 34% (all *P* < 0.05 vs. *Serca2* FF) lower labeling, respectively and a mosaic pattern of expression. With telemetric ECG surveillance, we observed no difference in basal heart rate, but the PR-interval was prolonged in *Serca2* KO mice: 49 ± 1 vs. 40 ± 1 ms (*P* < 0.001) in *Serca2* FF. During exercise, heart rate in *Serca2* KO mice was elevated to 667 ± 22 bpm, considerably less than 780 ± 17 bpm (*P* < 0.01) in *Serca2* FF. In isolated sinus node preparations, 2 mM Cs^+^ caused bradycardia that was equally pronounced in *Serca2* KO and *Serca2* FF (32 ± 4% vs. 29 ± 5%), indicating no change in the pacemaker current, *I*_f_. Disabling the Ca^2+^-clock with 2 μM ryanodine induced bradycardia that was less pronounced in *Serca2* KO preparations (9 ± 1% vs. 20 ± 3% in *Serca2* FF; *P* < 0.05), suggesting a disrupted Ca^2+^-clock. Mathematical modeling was used to dissect the effects of membrane- and Ca^2+^-clock components on *Serca2* KO mouse heart rate and sinus node action potential. Computer modeling predicted a slowing of heart rate with *SERCA2* downregulation and the heart rate slowing was pronounced at >70% reduction in *SERCA2* activity.

**Conclusions:** *Serca2* KO mice show a disrupted Ca^2+^-clock-dependent pacemaker mechanism contributing to impaired sinus node and atrioventricular node function.

## Introduction

**Figure 1:**
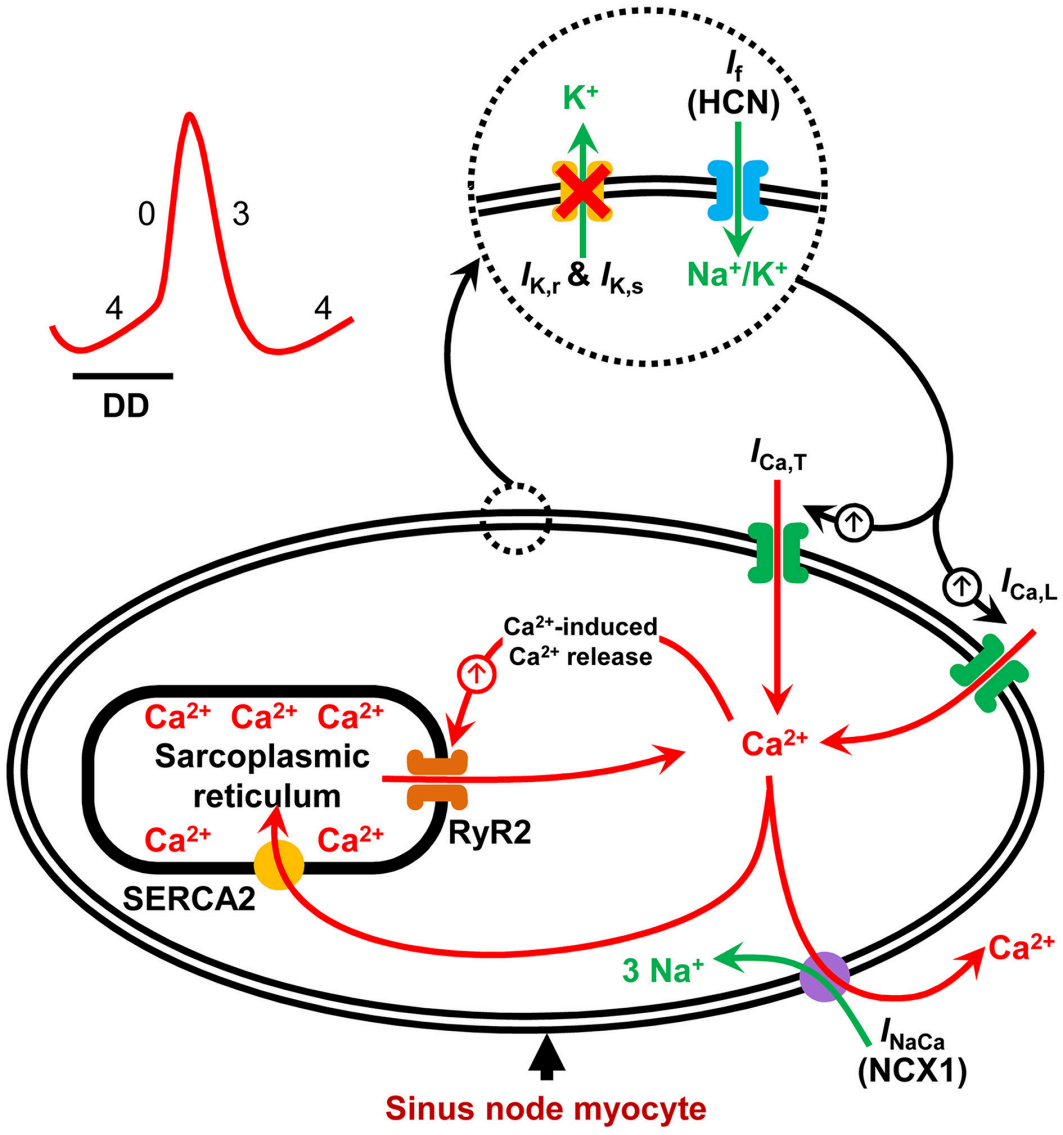
**Schematic illustration of pacemaker mechanisms in the sinus node myocyte**. Sinus node action potential is preceded by a slow diastolic depolarization (DD) which brings the membrane potential up to the threshold level for excitation. The DD is a result of synergistic interaction between the membrane voltage-clock and the subcellular Ca^2+^-clock. The membrane voltage-clock comprises plasma membrane bound, voltage-dependent ion channels and their corresponding ionic currents. At the beginning of the DD, there is voltage-dependent deactivation of outward K^+^ currents (*I*_K, r_ and *I*_K, s_) and activation of inward currents: hyperpolarization-activated cyclic nucleotide-gated (HCN) or funny current (*I*_f_), T-type Ca^2+^ current (*I*_Ca, T_) and L-type Ca^2+^ current (*I*_Ca, L_) amongst others. The Ca^2+^-clock contributes to sinus node DD through localized Ca^2+^ release from the sarcoplasmic reticulum via the ryanodine receptor (RYR2). Increased levels of cytosolic Ca^2+^ activates the electrogenic Na^+^–Ca^2+^ exchanger (NCX1) generating an inward current (*I*_NaCa_) that imparts a steep, exponential increase to the late phase of DD. SERCA2 refills the sarcoplasmic reticulum with Ca^2+^ and is hence crucial to sinus node pacemaking.

The sinus node is the dominant pacemaker of the heart, aptly placed in the roof of the right atrium. It consists of specialized pacemaker cells that spontaneously depolarize during diastole and rhythmically generate action potentials (Keith and Flack, 1907; Silverman et al., 2006; Dobrzynski et al., 2007). The diastolic depolarization results from the synergistic interaction between an ensemble of surface membrane ion channel currents and intracellular Ca^2+^ release signals. Together, they regulate the rate and rhythm of the spontaneous action potentials in the sinus node (Figure 1; Dobrzynski et al., 2007; Lakatta et al., 2010). The voltage- and time-dependent gating of the surface membrane channels works in a cyclic fashion (the membrane voltage-clock) and underlies the early phase of the diastolic depolarization. This phase involves a voltage-dependent deactivation of outward currents and activation of inward currents (Dobrzynski et al., 2013). The hyperpolarization-activated cyclic nucleotide-gated (HCN) or funny current (*I*_f_) is the main inward current, and thus a vital component of the voltage-clock and essential to pacemaking in the sinus node. Mice with a cardiac specific conditional knockout (KO) of the HCN4 channel have reduced *I*_f_ in sinus node cardiomyocytes and exhibit severe bradycardia (up to 50% reduction in heart rate; Baruscotti et al., 2011). Similarly, humans with HCN4 gene mutations exhibit sinus bradycardia (Dobrzynski et al., 2013).

In contrast to the early phase, the latter phase of the diastolic depolarization in sinus node pacemaking cells depends on localized, sarcoplasmic reticulum-generated, intracellular Ca^2+^ release via the ryanodine receptor, referred to as the intracellular Ca^2+^-clock. The released Ca^2+^ activates inward Na^+^-Ca^2+^ exchange, generating an inward current (*I*_NaCa_) by exchanging one Ca^2+^ for three Na^+^ (Figure 1; Bogdanov et al., 2006). Acute inhibition of *I*_NaCa_ is reported to stop pacemaking in single rabbit sinus node myocytes (Bogdanov et al., 2001). The localized release of Ca^2+^ from the sarcoplasmic reticulum is dependent upon the sarcoplasmic reticulum Ca^2+^-load which is regulated by the sarcoplasmic reticulum Ca^2+^-ATPase (SERCA) pump (Vinogradova et al., 2010). Inhibition of SERCA2 with cyclopiazonic acid induces a concentration-dependent suppression of spontaneous sinus node myocyte firing rate by up to 50% (Vinogradova et al., 2010; Yaniv et al., 2014).

We hypothesized that reduced *Serca2* in the sinus node disrupts the Ca^2+^-clock component of pacemaking and contributes to pacemaker dysfunction. This hypothesis was tested in a *Serca2* conditional KO mouse model in which cardiac specific *Serca2* excision can be induced at any age by exposure to tamoxifen.

## Methods

Homozygous, conditional cardiac specific *Serca2* KO and age-matched control (*Serca2* FF) mice were studied. Mice with *Serca2* downregulation in the heart were generated using the Cre-lox system conditional gene KO strategy as described previously by Andersson et al. (2009). At 12 weeks of age, male *Serca2* KO (*Serca2*^flox∕flox^ Tg(αMHC-MerCreMer); *n* = 22) and control (*Serca2*^flox∕flox^; FF, *n* = 20) mice were injected with 1 mg of tamoxifen intraperitoneally to induce *Serca2* excision in cardiomyocytes. All experiments were carried out 7 weeks after tamoxifen administration in accordance with the Norwegian National Committee for Animal Welfare Act, consistent with the NIH guidelines (NIH publication No. 85-23, revised 1996). SERCA2a abundance and expression pattern was investigated by Western blotting and immunohistochemistry (*n* = 10). Telemetry transmitters were implanted in one cohort of mice (*n* = 16) as previously described (Stokke et al., 2010). ECG was recorded under baseline conditions, during maximal exercise, and after a subsequent i.p. injection of adrenalin (0.5 mg/kg). *In vitro* electrophysiological investigation of the sinus node was carried out by recording extracellular potentials in isolated sinus node preparations (*n* = 16). Tissue preparations of Wistar rat hearts (*n* = 13), with intact sinus and AV nodes were used in some investigations.

Mathematical modeling of the mouse heart rate was performed using our biophysically detailed single pacemaker cell model of the mouse sinus node (Kharche et al., 2011). This robustly validated model describes the mouse sinus node electrophysiology based on a spectrum of membrane ionic currents (membrane voltage-clock), which are coupled to the intracellular Ca^2+^ dynamics (Ca^2+^-clock). The model code was implemented in C programming language, and was integrated using an implicit backward difference formula that provided a high accuracy of *O*(dt^5^), computational efficiency and unconditional numerical stability. The model code, including dependencies, is available from the authors on request. Standard library based code is available from the model repository, ModelsDB (https://senselab.med.yale.edu/modeldb/ShowModel.cshtml?model=141274). All data are expressed as mean ± s.e.m. and n refers to the number of hearts. Either an unpaired *t*-test or one-way ANOVA followed by Tukey's multiple comparisons post-test was conducted to determine significant differences. The difference was considered to be significant when *P* < 0.05. Detailed methodology is available in the Supplementary Material.

## Results

**Figure 2:**
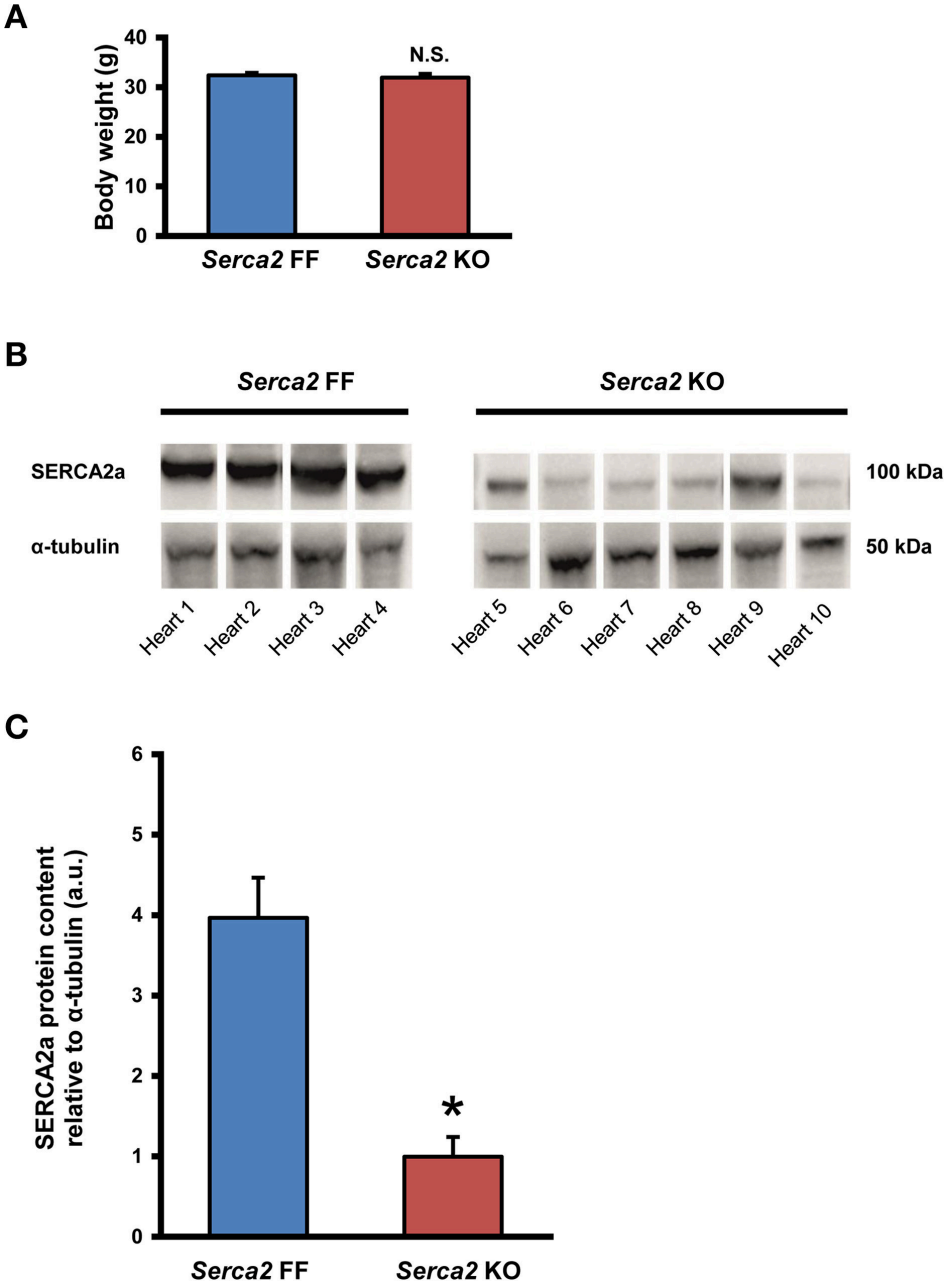
**SERCA2a expression in ventricular myocardium. (A)** Body weight of *Serca2* FF and *Serca2* KO mice. **(B)** Western blots of SERCA2a protein and α-tubulin expression in ventricular myocardium of *Serca2* FF (left; *n* = 4) and *Serca2* KO (right; *n* = 6) hearts. **(C)** Mean values of SERCA2a band density normalized to α-tubulin in *Serca2* FF and *Serca2* KO samples. ^*^P < 0.05 vs. *Serca2* FF.

The body weight of mice in the *Serca2* FF group and *Serca2* KO group were 32 ± 2 g (*n* = 12) and 32 ± 3 g (*n* = 14), respectively at 7 weeks after tamoxifen administration (Figure 2A).

### SERCA2A expression in the ventricular myocardium

**Figure 3:**
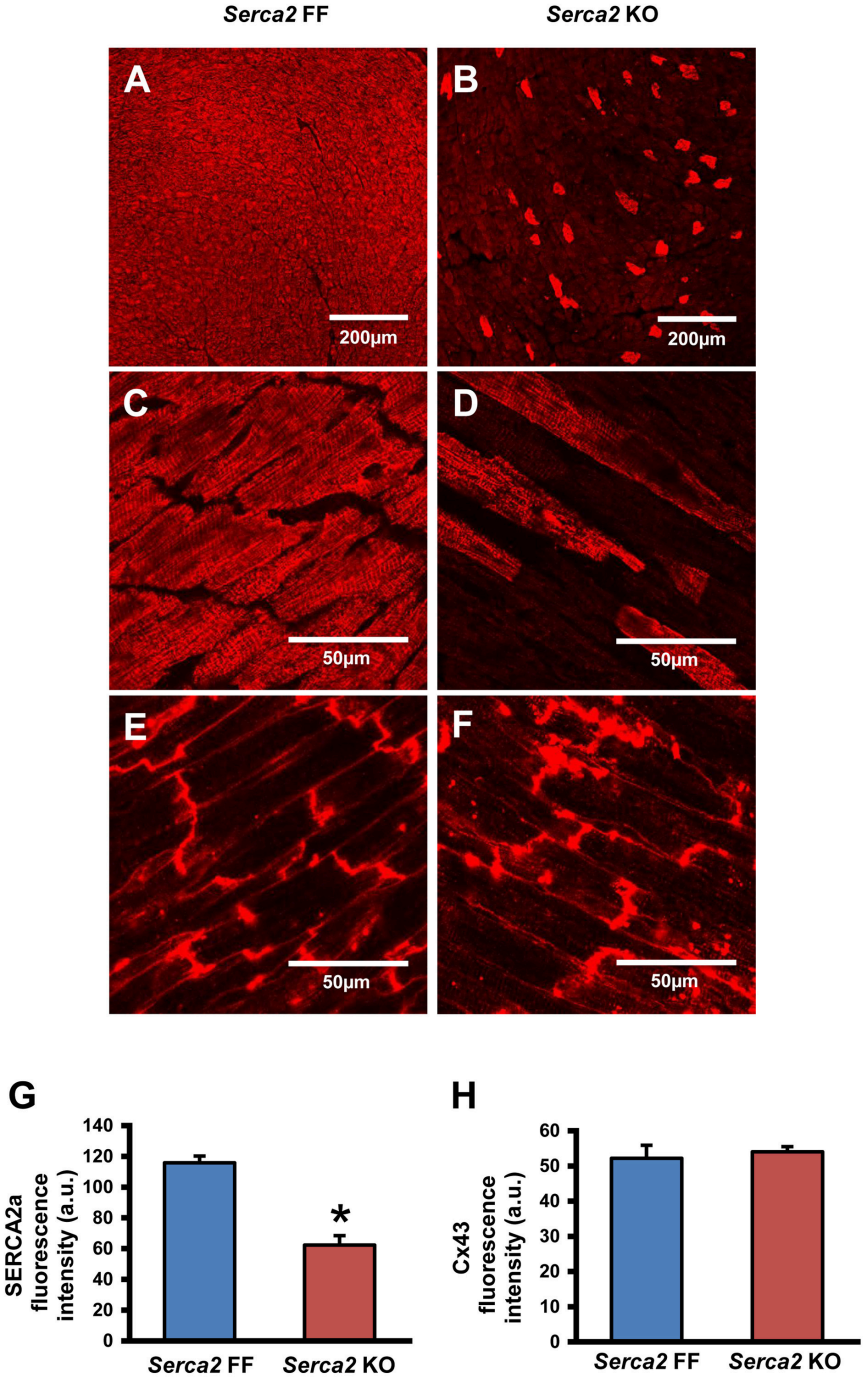
**Immunohistochemical detection of SERCA2a and Cx43 in ventricular myocardium. (A–D)** Representative immunofluorescence images of SERCA2a protein labeling (red signal) in ventricular myocardium of *Serca2* FF **(A,C)** and *Serca2* KO **(B,D)** hearts. **(E,F)**, high magnification images of Cx43 expression in ventricular myocardium of *Serca2* FF and *Serca2* KO hearts. **(G,H)** bar charts showing mean SERCA2a **(G)** and Cx43 **(H)** content in the ventricular myocardium of *Serca2* FF (blue bars; *n* = 4) and *Serca2* KO (red bars; *n* = 6) hearts. ^*^P < 0.05 vs. *Serca2* FF.

SERCA2a protein expression in the ventricular myocardium was investigated by Western blot and immunohistochemistry. Western blot analysis (Figure 2B) revealed that SERCA2a protein content normalized to α-tubulin content was reduced from 4.0 ± 0.5 a.u. (*n* = 4) in *Serca2* FF to 1.0 ± 0.3 a.u. (*n* = 6; *P* < 0.05) in *Serca2* KO ventricular myocardium (Figure 2C). The distribution and expression pattern of SERCA2a protein in ventricular myocardium was assessed by immunolabelling tissue sections with a SERCA2a antibody (Supplement Table 1). In ventricular myocytes, labeling occurred adjacent to the outer cell membrane, as well as in an internal striated pattern, consistent with earlier reports of SERCA2 protein expression in ventricular myocytes (Musa et al., 2002). Figures 3A–D shows the typical distribution of SERCA2a protein in ventricular cross sections of *Serca2* FF (*n* = 4; Figures 3A,C) and *Serca2* KO (*n* = 6; Figures 3B,D) mouse hearts. Similar patterns of expression were observed in all *Serca2* FF and *Serca2* KO tissue sections. At both low (Figure 3A) and high (Figure 3C) magnification, SERCA2a expression in the *Serca2* FF tissue was uniform with all cardiomyocytes expressing the SERCA2a protein. However, in the *Serca2* KO tissue sections, expression appeared reduced, mosaic-like and heterogeneous, i.e., some cells expressed and some did not (Figures 3B,D). The bright red signal in cardiomyocytes in Figures 3B,D represent ventricular myocytes expressing SERCA2a protein in the *Serca2* KO heart sections. The negative controls did not show fluorescence of any significance (Supplement Figure 1). Quantification of immunofluorescence measurements showed reduction in SERCA2a protein expression from 116 ± 4 a.u. (*n* = 4) in *Serca2* FF to 62 ± 6 a.u. (*n* = 6; *P* < 0.05) in *Serca2* KO sections (Figure 3G). The SERCA2a content measured by Western blot and immunohistochemistry in the individual heart samples was plotted (Supplement Figure 2). SERCA2a protein expression measured by either of the two techniques revealed a similar downregulation.

Connexin 43 (Cx43), a major gap junction connexin in the heart, was labeled in adjacent ventricular tissue sections and used as a marker of ventricular cardiomyocytes (Figures 3E,F). The quantified immunofluorescence of Cx43 labeling was 52 ± 4 a.u. (*n* = 4) in *Serca2* FF and 54 ± 2 a.u. (*n* = 6) in *Serca2* KO sections. The Cx43 protein expression remained unchanged (Figure 3H).

### Telemetry ECG

**Figure 4:**
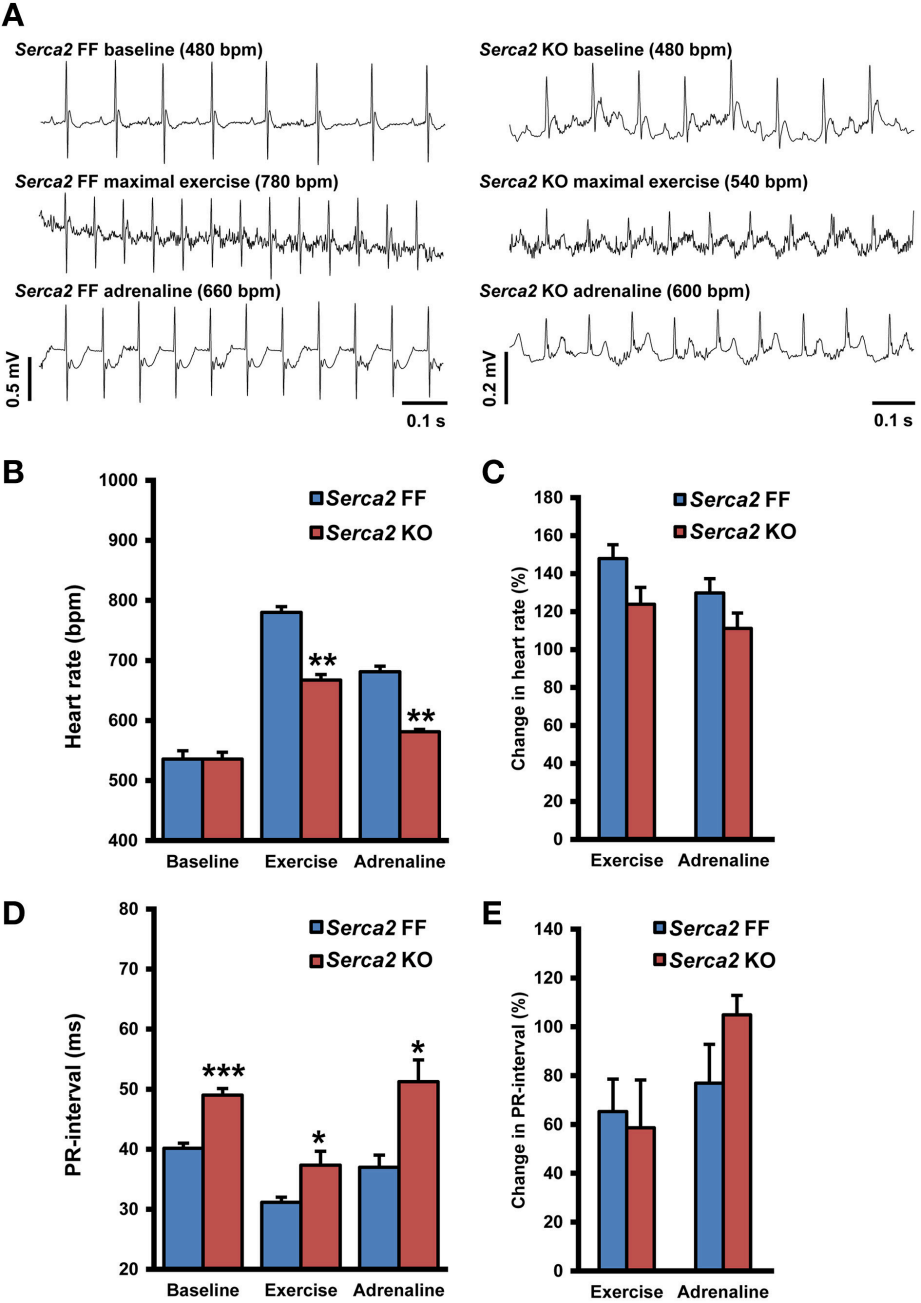
**Telemetry ECG parameters. (A)** Representative records of telemetry ECG in *Serca2* FF (left) and *Serca2* KO (right) mice under baseline conditions, maximal exercise, and after a subsequent i.p. injection of adrenaline (0.5 mg/kg). **(B)** Heart rate measurements in freely moving *Serca2* FF (blue bars) and *Serca2* KO (red bars) mice during baseline conditions, maximal exercise, and after a subsequent i.p. injection of adrenalin (0.5 mg/kg). **(C)** Change in heart rate during exercise and after adrenaline injection presented as a percentage of corresponding baseline values. **(D)** The PR-interval in *Serca2* FF (blue bars) and *Serca2* KO (red bars) mice during baseline conditions, maximal exercise, and after adrenaline administration. **(E)** Change in PR-interval presented as a percentage of corresponding baseline values. ^*^, ^**^, and ^***^ denote *P* < 0.05, *P* < 0.01, and *P* < 0.0001, respectively vs. corresponding Serca2 FF.

ECG was recorded in conscious animals with the aid of implanted telemetry electrodes (Figure 4A). The baseline heart rate measured in freely moving *Serca2* FF and *Serca2* KO animals was 536 ± 27 and 536 ± 31 beats per minute (bpm), respectively (both groups *n* = 8, Figures 4B,C). The treadmill test was performed as a gradual, stepwise increase in the running speed until exhaustion. *Serca2* FF achieved a maximal heart rate of 780 ± 17 bpm, significantly higher than the 667 ± 22 bpm achieved by *Serca2* KO (*P* < 0.01 vs. *Serca2* FF, Figures 4A–C). The distance traveled till exhaustion in *Serca2* FF was 117 ± 16 m and that in *Serca2* KO was 56 ± 6 m (*P* < 0.01). The maximum running speed was significantly higher in *Serca2* FF (18 ± 1 vs. 12 ± 1 m/min in *Serca2* KO, *P* < 0.05). Subsequently, animals were injected intraperitoneally (i.p.) with adrenaline (0.5 mg/kg bw) and at 10 min after adrenaline administration, the heart rates in *Serca2* FF and *Serca2* KO animals was 681 ± 9 and 581 ± 23 bpm, respectively (*P* < 0.01, Figures 4A–C). The ECG PR-intervals at baseline, during maximal exercise, and after a subsequent i.p. injection of adrenalin in *Serca2* FF was 40 ± 1, 31 ± 1, and 37 ± 2 ms and the corresponding values for *Serca2* KO were 49 ± 1 (*P* < 0.001 vs. *Serca2* FF), 37 ± 2 (*P* < 0.05 vs. *Serca2* FF) and 51 ± 4 ms (*P* < 0.05 vs. *Serca2* FF), respectively (Figures 4D,E).

### Sarcoplasmic reticulum Ca^2+^ and atrioventricular node

In rat right atrial preparations, the role of sarcoplasmic reticulum Ca^2+^ in sinus and atrioventricular (AV) node function was assessed *in vitro*. Ryanodine (2 μM) prolonged the sinus node intrinsic cycle length from 241.1 ± 9.63 ms (*n* = 13) to 369.8 ± 25.4 ms (*n* = 10; *P* < 0.001). The tissue was overdrive paced and the atrio-His (AH) interval (a measure of conduction through the AV node) and the AV node Wenckebach cycle length were measured. Ryanodine (2 μM) prolonged the AH-interval from 47 ± 6.1 ms to 59 ± 7.7 ms (*P* ≤ 0.01) and the AV node Wenckebach cycle length from 161.5 ± 11.7 ms to 205.1 ± 22.7 ms (*P* ≤ 0.05).

### Sinus node pacemaking in *Serca2* KO mice

**Figure 5:**
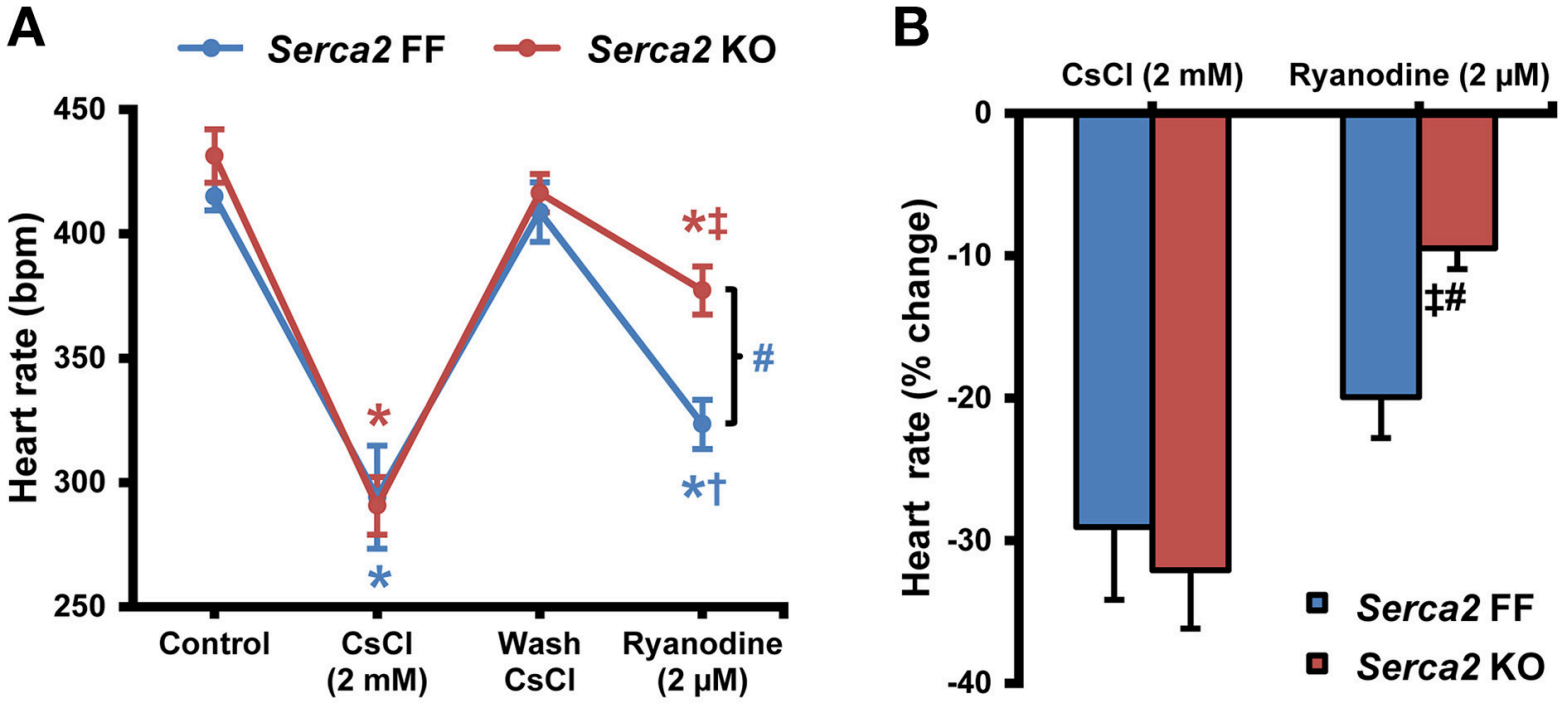
**Sinus node pacemaking in ***Serca2*** FF vs. ***Serca2*** KO mice. (A)** Change in heart rate of isolated sinus node preparations from *Serca2* FF and *Serca2* KO in response to treatment with 2 mM CsCl and 2 μM ryanodine. **(B)** Percentage reduction of heart rate *in vitro* induced by CsCl (2 mM) and ryanodine (2 μM) treatment. The CsCl (2 mM) and ryanodine (2 μM) measurements are presented as percentage of control and wash CsCl values, respectively. *Serca2* FF data are shown in blue and *Serca2* KO data are shown in red color. ^*^, †, and ‡ represent *P* < 0.05 vs. control, wash CsCl and CsCl (2 mM) respectively. Blue symbols are used for *Serca2* FF and red for *Serca2* KO data. ^#^represents *P* < 0.05 vs. *Serca2* KO ryanodine response (*Serca2* FF, *n* = 8 and *Serca2* KO, *n* = 8).

The intrinsic beating/heart rate measured in isolated sinus node preparations at 37°C was 415 ± 16 bpm (*n* = 8) in *Serca2* FF and 431 ± 30 bpm (*n* = 8) in *Serca2* KO (Figure 5A) tissues. The response to pharmacological block of the membrane- and Ca^2+^-clock was measured by separately treating tissues with 2 mM Cs^+^ (for *I*_f_ block) and 2 μM ryanodine (for disabling Ca^2+^ release via the ryanodine receptor). In the presence of Cs^+^, the sinus rate reduced to 294 ± 59 bpm (*n* = 8) in *Serca2* FF and 291 ± 33 bpm (*n* = 8) in *Serca2* KO (Figure 5A) mice. The response to ryanodine was different in *Serca2* KO compared to *Serca2* FF mice. The sinus rate dropped to 323 ± 26 bpm (*n* = 7) in *Serca2* FF mice whereas in *Serca2* KO mice, the drop in sinus rate was to 377 ± 26 bpm (*n* = 7; Figures 5A,B). In other words, the effect of ryanodine was less pronounced in *Serca2* KO tissue in comparison with *Serca2* FF tissue.

### SERCA2 downregulation in sinus node

**Figure 6:**
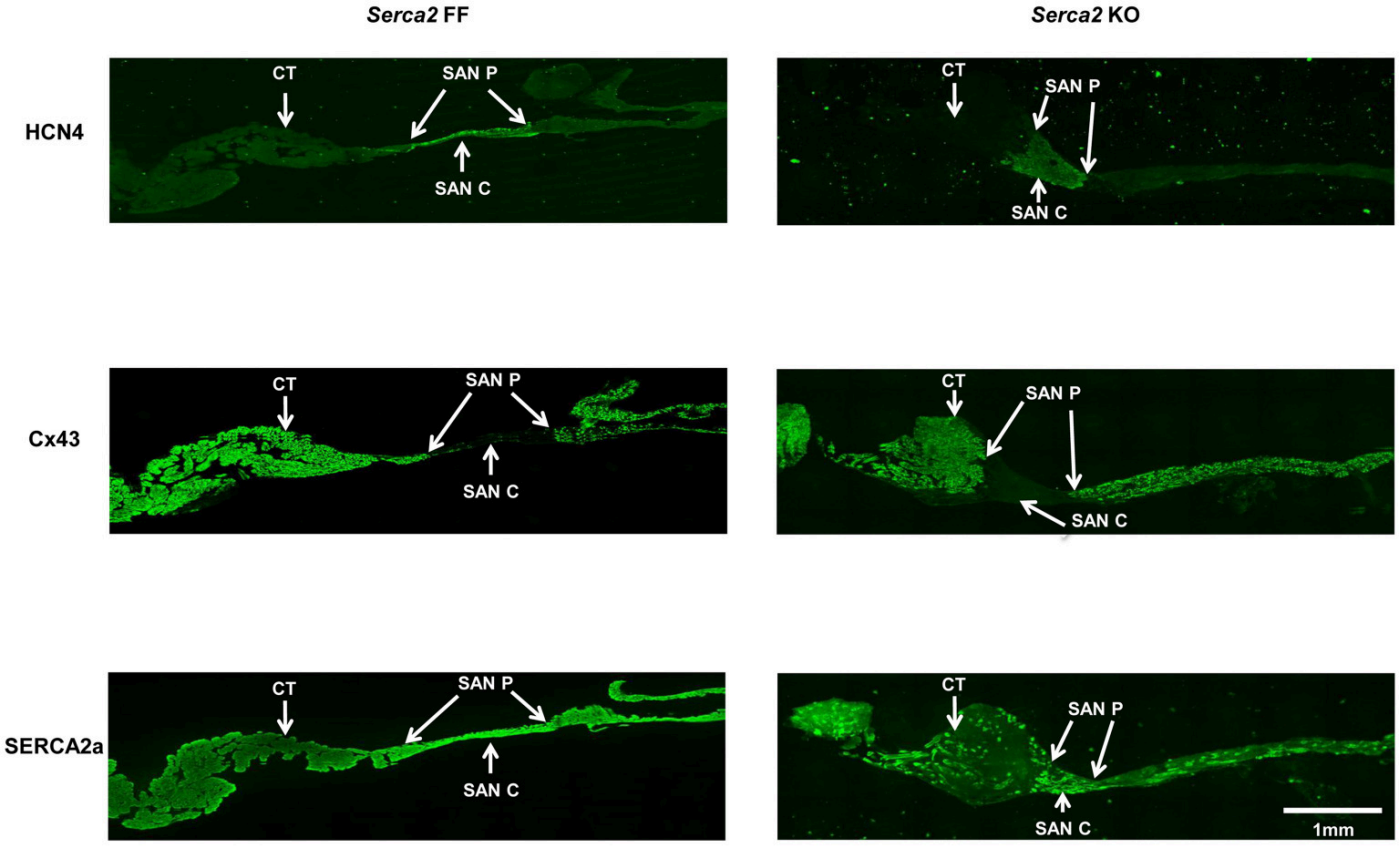
**Immunolabelling of HCN4, Cx43, and SERCA2 in right atrial sections from ***Serca2*** FF and ***Serca2*** KO hearts**. Top row, right atrial sections from *Serca2* FF (left) and *Serca2* KO (right) hearts showing HCN4 labeling (green signal) in the sinus node periphery (SAN P) and center (SAN C). Middle row, connexin 43 (Cx43) labeling (green signal) in the right atrium. Bottom row, right atrial sections showing SERCA2a labeling (green signal) throughout the tissue section in *Serca2* FF (left) and a mosaic pattern of SERCA2a labeling in *Serca2* KO (right). *Abbreviations*: CT, *crista terminalis*; SAN P, sinus node periphery; SAN C, sinus node center.

**Figure 7:**
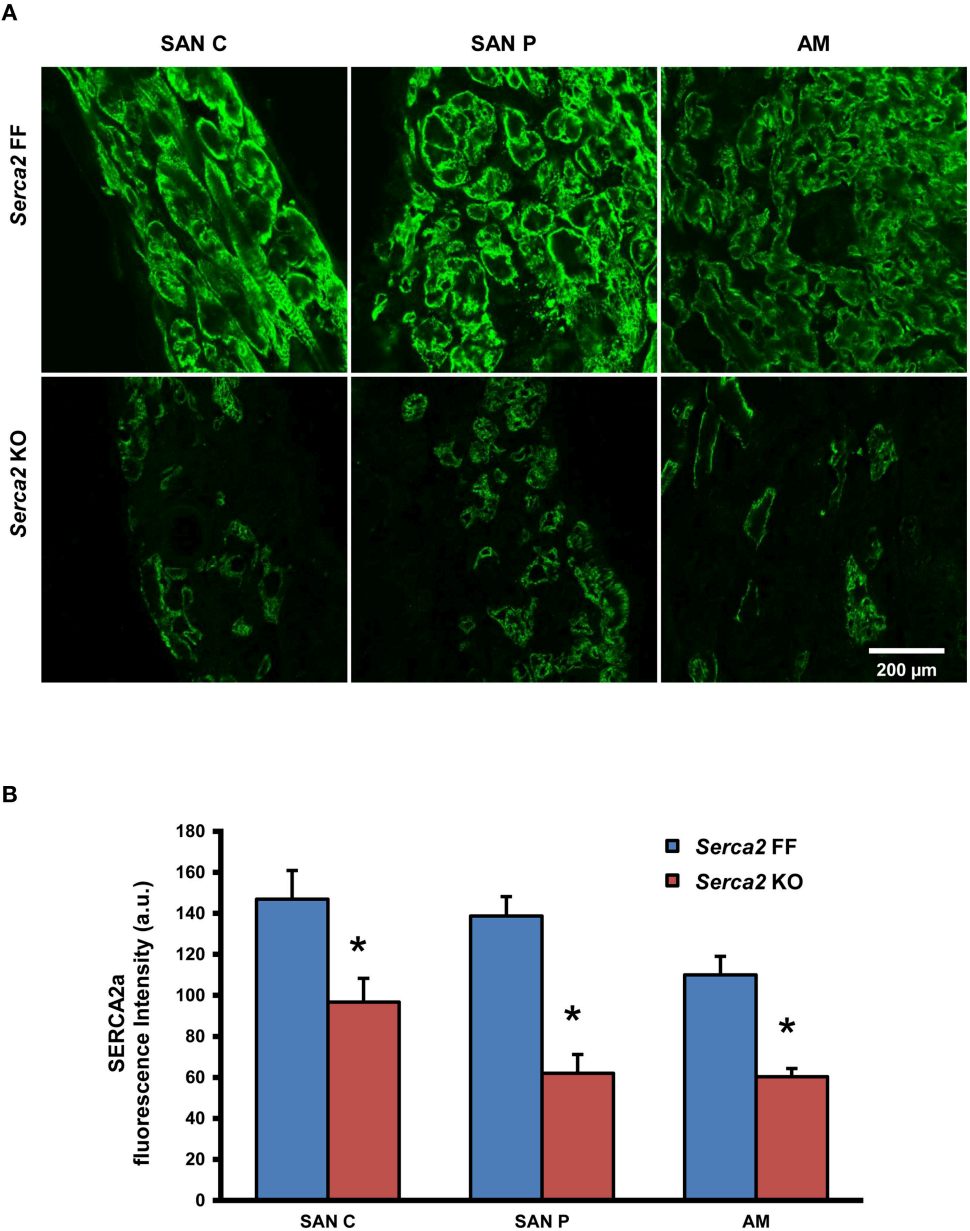
**SERCA2a protein expression in the right atrium and sinus node. (A)** Representative high magnification images showing SERCA2 labeling (green signal) in the sinus node center (SAN C), sinus node periphery (SAN P) and atrial muscle (AM) of *Serca2* FF (top row) and *Serca2* KO (bottom row) right atrial tissue sections. **(B)** SERCA2a fluorescence intensity in the sinus node center (SAN C), sinus node periphery (SAN P) and atrial muscle (AM) of *Serca2* FF (blue bars) and *Serca2* KO (red bars) tissue sections. ^*^ denotes *P* < 0.05 vs. corresponding *Serca2* FF tissue values.

Histological staining with Masson's trichrome was performed in tissue sections that were cut perpendicular to the *crista terminalis* of *Serca2* FF and *Serca2* KO right atrial tissue. The morphology and architecture of the sinus node was not affected in *Serca2* KO mice (data not shown). The histology images were used to locate the sinus node and identify suitable sections for labeling with immunofluorescent markers. HCN4 was used as the positive marker to delineate sinus node myocytes and Cx43 was used to label atrial muscle (Liu et al., 2007). Typical low magnification images of HCN4 and Cx43 are shown in Figure 6 (top and middle row). Adjacent sections labeled for SERCA2a are shown in Figure 6 (bottom row). Representative high magnification images of the sinus node center, sinus node periphery and atrial muscle are shown in Figure 7A. The SERCA2a expression pattern in these sections was similar to that seen in sections from the ventricular myocardium: in the *Serca2* FF sinus node center, sinus node periphery and atrial muscle, SERCA2a protein was ubiquitously expressed in all myocytes (Figure 7A, top row). In contrast, in the *Serca2* KO sections, SERCA2a expression was reduced and mosaic-like in all three regions (Figure 7A, bottom row). The quantified fluorescence measurements in *Serca2* FF sections were 147 ± 14, 139 ± 10, and 110 ± 9 a.u. (*n* = 4) in the sinus node center, sinus node periphery and atrial muscle, respectively (Figure 7B). In *Serca2* KO sections, fluorescence measurements were reduced to 97 ± 12, 62 ± 9, and 60 ± 4 (*n* = 6; all *P* < 0.05 vs. corresponding *Serca2* FF values). HCN4 and Cx43 expression patterns were similar in *Serca2* FF and *Serca2* KO sections (Supplement Figures 3, 4).

### Mathematical modeling of mouse sinus node action potential

**Figure 8:**
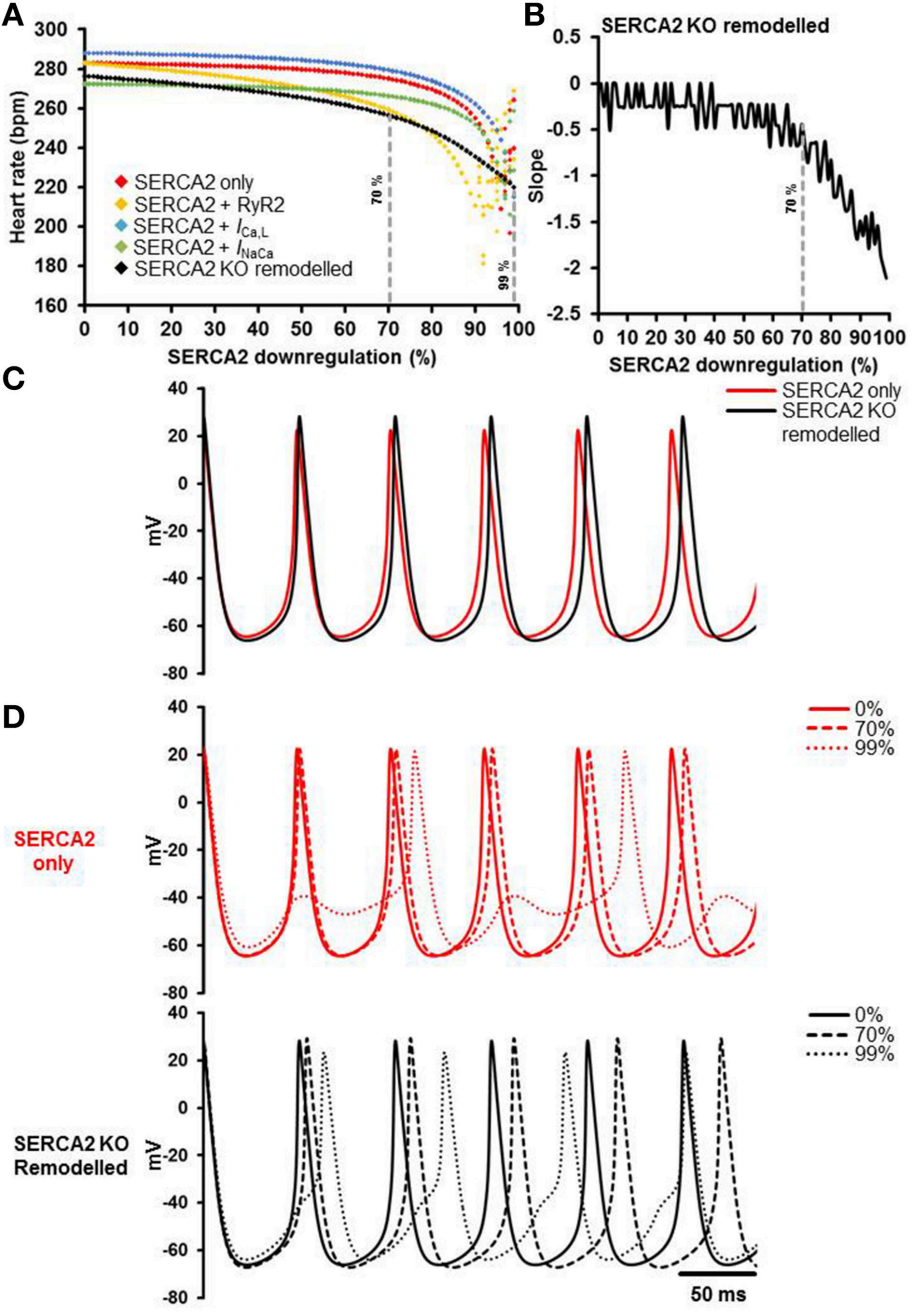
**Simulation of the effects of SERCA2 downregulation on the sinus node action potential. (A)** Relationship between SERCA2 downregulation (0–99% reduction) and heart rate (bpm) under various conditions. Red symbols represent heart rates with changes to SERCA2 only (i.e., with all other parameters unchanged). Yellow, blue and green symbols represent introduction of changes to RyR2 (proportional to SERCA2), *I*_Ca, L_ (1.3 fold increase), and *I*_NaCa_ (2 fold increase), respectively. The combined effect of all changes is represented in black (SERCA2 KO remodeled). **(B)** Slope of the SERCA2 KO remodeled curve. **(C)** Action potential profiles of SERCA2 only (red) and SERCA2 KO remodeled (black) at 0% SERCA2 downregulation are shown. **(D)** Typical action potential profiles under SERCA2 only in red (top) and SERCA2 KO remodeled in black (bottom) are shown. Solid, dashed and dotted lines represent 0, 70, and 99% SERCA2 downregulation respectively. Modeling data generated by SK.

Mathematical modeling was used to dissect the role of SERCA2 in sinus node pacemaking and to gain mechanistic insights. The results of the simulations are presented in Figure 8. The relationships between SERCA2 downregulation (0–99%) and heart rate under various conditions is shown in Figure 8A. Pacemaking in the sinus node, measured as heart rate, was significantly more erratic at low levels of SERCA2 (99% downregulation) than at physiological levels of SERCA2 (0% downregulation) (red symbols, Figure 8A). At physiological values of SERCA2, heart rate of 283 bpm was observed. Downregulation of RyR2 channel (proportional to SERCA2 downregulation) did not affect the heart rate at 0% SERCA2 downregulation; however, it increased the arrhythmic region at >90% SERCA2 downregulation (yellow symbols, Figure 8A). The increase of L-type Ca^2+^ current (*I*_Ca, L_) caused an overall increase of heart rate at all levels of SERCA2 (blue symbols, Figure 8A). The heart rate at 0% SERCA2 downregulation was 287 bpm. On the other hand, increasing the Na^+^-Ca^2+^ exchanger current (*I*_NaCa_) resulted in lowering of heart rate: 272 bpm at 0% SERCA2 downregulation (green symbols, Figure 8A). When all the above alterations were implemented simultaneously to emulate the experimental observations, i.e. enhanced Ca^2+^ flux through the L-type Ca^2+^ channel and the Na^+^-Ca^2+^ exchanger in *Serca2* KO mouse (Andersson et al., 2009), the resultant heart rate (black symbols, Figure 8A), was lower than in the case without remodeling (red symbols, Figure 8A). The slope of the remodeled heart rate curve is shown in Figure 8B. It was observed that the slope was small for low levels of SERCA2 downregulation and numerically large at >70% downregulation.

Action potential profiles of SERCA2 only (red) and SERCA2 KO remodeled (black) at 0% SERCA2 downregulation is shown in Figure 8C. Corresponding action potential profiles at 0, 70, and 99% SERCA2 downregulation are shown in Figure 8D. Without any remodeling, beating/heart rates measured at 0 and 70% SERCA2 downregulation were 283 bpm (red solid line, Figure 8D) and 275 bpm (red dashed line, Figure 8D) respectively; however, at 99% downregulation the beating/heart rate was substantially reduced to 220 bpm (red dotted line, Figure 8D) and the region with >95% SERCA2 downregulation was arrhythmic. Under remodeled conditions, the corresponding heart rates were 276 bpm (black solid line, Figure 8D), 256 bpm (black dashed line, Figure 8D) and 220 bpm (black dotted line, Figure 8D) respectively. In the remodeled case at 99% SERCA2 downregulation, the maximum diastolic potential was less negative (by ~3 mV), the action potential upstroke was biphasic and the heart rate was substantially bradycardic (black dotted line, Figure 8D).

## Discussion

We have investigated the consequence of substantial *Serca2* downregulation on the sinus node of the adult mouse heart. Cardiomyocyte-specific excision of *Serca2* in adult mice resulted in reduced SERCA2a protein abundance in the sinus node, atrial muscle and ventricular muscle. *In vivo*, baseline heart rate was not affected; however, during exercise and in the presence of adrenaline, heart rate was lower than *Serca2* FF. *In vitro*, the intrinsic sinus node beating rate remained unaltered in *Serca2* KO. Separate assessment of the voltage- and Ca^2+^-clock components of sinus node pacemaking revealed an unaltered voltage-clock and a diminished Ca^2+^-clock. Our *Serca2* KO mouse model provides novel information on the importance of SERCA2a, and thus the sarcoplasmic reticulum Ca^2+^ clock, in the sinus node pacemaking mechanism.

In our study, we have demonstrated that SERCA2a downregulation in the sinus node of the adult mouse heart can be achieved by relying on the α-MHC promoter to drive the expression of the Cre-recombinase enzyme to cause cardiac specific *Serca2* deletion. The reduction in SERCA2a protein content occurs in the sinus node, as well as atrial and ventricular tissues, suggesting Cre expression in all of these tissues. α-MHC is a contractile protein and mRNA corresponding to α-MHC has been reported in the ventricular myocardium, as well as in the atrium and sinus node (Nakao et al., 1997; Tellez et al., 2011). Thus, Cre-recombinase would be abundantly expressed in these tissues resulting in *Serca2* deletion. Our observations are consistent with a recent study of a cardiac specific HCN4 gene KO mouse model wherein the α-MHC promoter linked Cre-Lox system of gene deletion induced substantial downregulation of HCN4 protein in the sinus node (Baruscotti et al., 2011).

In the ventricular myocardium, we found that SERCA2a protein expression was reduced by ~75% at 7 weeks after tamoxifen administration. The mosaic pattern of SERCA2a expression observed in *Serca2* KO heart tissue was an interesting, yet unexpected finding of this study. It should be borne in mind that, conditional gene KO techniques based on excision of lox-P-flanked DNA segments by Cre-recombinase have some inherent pitfalls (Schmidt-Supprian and Rajewsky, 2007). LoxP-flanked target genes differ in their sensitivity to Cre-facilitated recombination and Cre can damage genomic DNA. Also, the efficiency of gene KO using Cre-loxP technology is tamoxifen dose-dependent (Schmidt-Supprian and Rajewsky, 2007; Bersell et al., 2013). Tamoxifen administration to mice expressing MerCreMer protein is known to induce severe, transient dilated cardiomyopathy that is accompanied by transient reduction in SERCA2 and phospholamban mRNA (Koitabashi et al., 2009). Thus, data obtained immediately after tamoxifen induced gene inactivation, is often unreliable. In the present study, hearts were harvested at 7 weeks post tamoxifen administration, giving sufficient time for the transient cardiomyopathy to resolve (Koitabashi et al., 2009). Western blot data showed substantial reduction in SERCA2a protein content in the left ventricular myocardium, in agreement with an earlier study of this animal model (Andersson et al., 2009). Several other studies have administered tamoxifen as 4–5 injections on successive days at a safe dose (1–2 mg/day) (Danielian et al., 1998; Andersson et al., 2009; Hougen et al., 2010; Baruscotti et al., 2011). Our results indicate that a single dose of tamoxifen (1 mg/day) is sufficient to induce efficient Cre-recombination and substantial *Serca2* downregulation. However, the downregulation is heterogeneous, unlike the homogeneous *Serca2* downregulation previously observed by us in ventricular tissue for the single dose tamoxifen injection (Hougen et al., 2010). The single tamoxifen dose (1 mg/day) may be on the threshold limit for efficient MerCreMer-activation (as opposed to four injections). Thus, some cells might not be exposed to an adequate concentration, giving rise to a mosaic pattern of downregulation as seen elsewhere (Hayashi and McMahon, 2002).

Isolated ventricular myocytes of *Serca2* KO mice show significantly lower sarcoplasmic reticulum Ca^2+^ content (Swift et al., 2008, 2012; Andersson et al., 2009; Stokke et al., 2010). Given that we used an identical mouse model (albeit with one tamoxifen injection instead of four), and that the SERCA2 protein is substantially downregulated, it is reasonable to expect a significant degree of reduction in sarcoplasmic reticulum Ca^2+^ content in sinus node myocytes. Reduced sarcoplasmic reticulum Ca^2+^ content decreases the open probability of the ryanodine receptor and the occurrence of localized Ca^2+^ release events that govern the late phase of the diastolic depolarization in the sinus node (Bogdanov et al., 2006). Also, the kinetics of the sarcoplasmic reticulum Ca^2+^ pumping by SERCA2 is known to regulate sinus node beating rates (Vinogradova et al., 2010). *In vitro*, pharmacological blockade of either the ryanodine receptor with ryanodine or the SERCA2 pump with cyclopiazonic acid, inhibits the sinus node beating rate (Rigg and Terrar, 1996; Vinogradova et al., 2010; Yaniv et al., 2014). One might then ask why the *Serca2* KO mice baseline heart rates measured *in vivo* (536 ± 31 vs. 536 ± 27 bpm in *Serca2* FF) and sinus node beating rates measured *in vitro* (431 ± 30 vs. 415 ± 16 bpm in *Serca2* FF tissues) were not different from those in *Serca2* FF? In the present study, although SERCA2a is significantly downregulated (by 70–75%) in *Serca2* KO hearts, some degree of residual SERCA2a activity will exist and is likely to contribute to the maintenance of baseline heart/sinus rates. Previously, we have observed baseline bradycardia in *Serca2* KO with >95% SERCA2a downregulation and have demonstrated that very low SERCA levels are capable of partially refilling the sarcoplasmic reticulum (Andersson et al., 2009; Louch et al., 2010). The residual SERCA2a activity is however, below optimal during exercise and in the presence of adrenaline resulting in smaller heart rate increases. Additionally, it is possible that compensatory mechanisms may have come into play during the 7 week period to allow for the *Serca2* downregulation. Blocking *I*_f_ slowed pacemaking that was equally pronounced in *Serca2* KO and control mice (Figure 4) and hence it is unlikely that *I*_f_ compensates for the loss of the Ca^2+^-clock. It is possible that some other compensatory mechanism exists. Pacemaking in the sinus node is also dependent on the voltage-dependent deactivation of outward currents: the rapid and slow delayed rectifier K^+^ currents (*I*_K, r_ and *I*_K, s_) and activation of inward currents such as the L- and T-type Ca^2+^ currents (*I*_Ca, L_ and *I*_Ca, T_), *I*_NaCa_, tetrodotoxin-sensitive Na^+^ current (*I*_Na_) and sustained inward current (*I*_st_), amongst others (Dobrzynski et al., 2013; Logantha et al., 2014; Morris and Kalman, 2014).

Studies on isolated ventricular myocytes of *Serca2* KO mice have shown larger *I*_Ca, L_ and *I*_NaCa_ density, indicating a greater Ca^2+^-influx and extrusion across the plasma membrane (Andersson et al., 2009). Using mathematical modeling, we evaluated the consequences of increased *I*_Ca, L_ and *I*_NaCa_ on the heart rate of *Serca2* KO. Downregulation of SERCA2 alone caused a reduction in heart rate that was pronounced at >70% SERCA2 downregulation. At >95% SERCA2 downregulation, pacemaking was arrhythmic, i.e., non-periodic. When SERCA2 downregulation was coupled with a proportional reduction of Ca^2+^ flux through the RyR2, the region of arrhythmic pacemaking was increased. This is in agreement with our previous work, as well as work by others (Kharche et al., 2011; Maltsev and Lakatta, 2013). As the membrane- and Ca^2+^-clocks in our model are based on extensive experimental data, it was possible to simulate the phenomenon of how SERCA2 alterations affect heart rate. In the sinus node model, *I*_Ca, L_ regulates the upstroke of the action potential and the heart rate (Kharche et al., 2011). On the other hand, *I*_NaCa_ mainly provides calcium homeostasis. Therefore, in the current study, increasing *I*_Ca, L_ increased the heart rate and *I*_NaCa_ augmentation resulted in lower heart rate measurements due to a lower level of cytosolic Ca^2+^. Simultaneously altering multiple parameters (conductance of SERCA2, RyR2, *I*_Ca, L_, and *I*_NaCa_) does not result in a straightforward overlay of the outcomes (i.e., heart rates) because the cell model is complex and non-linear (Kharche et al., 2009). Implementing the remodeling as a simultaneous change of function in RyR2, *I*_Ca, L_, and *I*_NaCa_ resulted in reduced heart rates. The heart rate changes minimally upto 70% SERCA2 downregulation; however, at >70% downregulation the reduction in heart rate is much pronounced. This is consistent with our experimental observations and it is likely that similar compensatory remodeling occurs in the sinus node which helps maintain baseline heart rate *in vivo* and *in vitro*. Although, mathematical models cannot encompass all the physiological processes in a sinus node cell, our model is capable of dissecting the important mechanisms that regulate pacemaking (See Model Limitations in Supplementary Material).

*In vivo*, with the sympathetic innervation intact, the resting heart rates of both *Serca2* KO and *Serca2* FF mice are higher than that observed *in vitro* in isolated right atrial preparations. In our study, *Serca2* KO mice with 75% reduction in left ventricular SERCA2a protein content show a smaller increase in heart rate vs. *Serca2* FF in response to exercise and adrenaline injection. This is consistent with the smaller increase in heart rate in response to isoproterenol observed in *Serca2* KO mice with >95% SERCA2a downregulation (Boardman et al., 2014). Catecholamine mediated acceleration of sinus node pacemaking is crucially dependent on the Ca^2+^-clock (Gao et al., 2010; Lakatta et al., 2010; Liu et al., 2011).

The ECG PR-interval, a measure of AV node conduction, was significantly prolonged in *Serca2* KO mice. In the present study, we also show that ryanodine, by disrupting sarcoplasmic reticulum Ca^2+^ releases, prolongs the AH-interval and the Wenckebach cycle length in isolated rat AV node preparations. This is consistent with previous work from our laboratory that showed that ryanodine increases the spontaneous cycle length of intact AV node preparations (Nikmaram et al., 2008). Additionally, both ryanodine and SERCA inhibitors (thapsigargin and cyclopiazonic acid) prolong the spontaneous cycle length in isolated AV node myocytes and the beating rate is therefore dependent on sarcoplasmic reticulum function (Ridley et al., 2008; Cheng et al., 2011). The prolonged PR-interval in *Serca2* KO mice is indicative of first degree AV-block and points at the important role for sarcoplasmic reticulum Ca^2+^ in AV node function.

## Clinical perspectives and future applications

SERCA2 is an important Ca^2+^ handling protein in the sinus node. *Serca2* expression as well as protein abundance is significantly altered in the diseased or aged sinus node (Tellez et al., 2011; Benoist et al., 2014). It has been long established that SERCA2a expression and activity is decreased in human heart failure of most etiologies and SERCA2a is a promising target for gene therapy in heart failure (Unverferth et al., 1988; Mercadier et al., 1990; Hasenfuss et al., 1994; Bers et al., 2003; Hayward et al., 2014). However, it has not been clear whether *Serca2* loss is a basis for or a result of sinus node disease and heart failure. Here we show for the first time that *Serca2* downregulation can cause pacemaker dysfunction, which might contribute to further deterioration in an already failing heart. The *Serca2* KO mouse developed for this study is a valuable tool in the investigation of the role of the sarcoplasmic reticulum in sinus node and AV node function. Previously, such investigations were limited by the use of pharmacological tools to block the SERCA2 pump or ryanodine receptor. The *Serca2* KO mouse offers a suitable platform for mechanistic studies at the level of the single cell, tissue and the whole animal.

## Author contributions

MS, IS, OS, SL, and HD conceived the project, secured funding and planned the experiments. MS performed telemetry ECG monitoring and analysis. SL carried out the *in vitro* electrophysiology experiments and collected tissue samples for Western blot and immunohistochemistry. AA performed Western blot. SL, SP, and HD were responsible for cryosectioning, histology staining, immunolabelling, immunofluorescence quantification and analysis. YS performed the experiments on the isolated rat atrioventricular node. AA, SP, and YS were supervised by HD. SL provided the experimental data input to SK for the mathematical modeling. SK designed and performed mathematical modeling and interpreted the simulation results. SL generated the figures and prepared the first draft of the manuscript. All authors contributed to the revision of manuscript and have approved the manuscript.

## Funding

SL was awarded a travel grant by the Physiological Society, London and financial support by the University of Oslo for a visit to Oslo to carry out electrophysiological investigations and collect tissue samples. He also received salary support from the British Heart Foundation (Programme Grant RG/11/18/29257).

### Conflict of interest statement

The authors declare that the research was conducted in the absence of any commercial or financial relationships that could be construed as a potential conflict of interest.
