# Supplementary material for: Ca2+-Clock-Dependent Pacemaking in the Sinus Node Is Impaired in Mice with a Cardiac Specific Reduction in SERCA2 Abundance
Source: Front Physiol. 2016 Jun 2;7:197. doi: 10.3389/fphys.2016.00197 (PMC4889599; doi:10.3389/fphys.2016.00197)
Supplement: Supplementary file 1 [file Presentation1.pdf]

SUPPLEMENTARY MATERIAL

Supplementary Methods: *Serca2* FF and *Serca2* KO mice

Telemetric ECG surveillance

ECGs were recorded in freely moving mice carrying telemetry transmitters (Physiotel ETA-F10, Data Sciences International, St. Paul, MN, USA). The implant procedure was performed as previously described,[1] 6 weeks after the injection with tamoxifen. Anaesthesia was induced with xylazine hydrochloride and ketamine hydrochloride i.p. combined with isoflurane inhalation (1-4%). A subcutaneous injection of buprenorphine was given towards the end of the procedure for postoperative analgesia. The transmitters were placed subcutaneously in the dorsal thoracolumbar region, and a stable midline position was ensured by ligatures to the dorsal muscles. Leads were placed in the upper left and lower right pectoral region and attached to the pectoral muscles by ligatures. Animals were allowed to recover from surgery for 7 days before ECG recordings. Recordings were analysed for heart rate as well as ECG parameters during baseline conditions, during maximal exercise, and after a subsequent i.p. injection of adrenalin (0,5mg/kg). The treadmill test was performed as a gradual, stepwise increase in the running speed until exhaustion. Recordings immediately prior to exhaustion were taken as the maximal exercise. Analysis was performed by manual scanning of all ECGs to identify areas with stable recordings for at least 30 s in all conditions. Heart rate and PR-interval measurement was performed by semi-automatic analysis using Ponemah Software (Data Sciences International, St. Paul, MN, USA).

Tissue preparation for electrophysiology

Animals were killed by cervical dislocation and the hearts were quickly removed and placed in Tyrode's solution of the following composition: 120.3 mM NaCl, 4.0 mM KCl, 1.3 mM $MgSO_4$, 1.2 mM NaH_2PO_4, 1.2 mM $CaCl_2$, 25.2 mM $NaHCO_3$ and 11.0 mM glucose. The solution was constantly bubbled with 95% O_2 and 5% CO_2 gas mixture to maintain pH at 7.4. The right atrium was quickly dissected in oxygenated Tyrode's solution. Throughout dissection, care was taken to keep all incisions as far away as possible from the sinus node and the isolated preparations typically had the entire sinus node, right atrial appendage, intercaval region and sections of the superior and inferior vena cava intact. All excess fat tissue in the vicinity of the sinus node was trimmed off and the sinus node preparations were quickly transferred to a tissue chamber for electrophysiological investigations. Tyrode's solution was circulated through the tissue chamber at a rate of 10 ml/min and the temperature in the vicinity of the tissue was constantly maintained at 37±0.5°C. The tissue was allowed to equilibrate for at least 20 min before carrying out any pharmacological interventions.

In vitro electrophysiology

Intrinsic sinus node beating rates were determined *in vitro*, by recording extracellular potentials from the endocardial surface of the isolated right atrial preparations. Potentials were captured using custom made stainless steel pin (100 µm diameter) electrodes. Data were continuously recorded onto a PC via a PowerLab high-performance data acquisition system and LabChart 7 software (both AD Instruments, UK). Peaks in the electrogram corresponding to the sinus node beats were detected using the ECG analysis module in PowerLab 7. Cycle length values were calculated by averaging individual beat-beat interval measurements of 500 consecutive beats.

The effect of *Serca2* KO on sinus node pacemaking was investigated pharmacologically in isolated sinus node preparations obtained from *Serca2* KO (n=7) and *Serca2* FF (n=7) mouse hearts. The beating/heart rate of sinus node preparations were continuously recorded during pharmacological treatments. A stable baseline recording of the intrinsic sinus node beating rate was first obtained and the circulating solution was then changed to Tyrode's solution containing 2 mM CsCl to inhibit the I_f which is the major component of the membrane-voltage clock.[2] After 20 min, the tissue was washed in normal Tyrode's solution for 30 min to allow sinus rates to recover to baseline levels. The spontaneous Ca^{2+} release from the sarcoplasmic reticulum via the ryanodine receptor (Ca^{2+}-clock mechanism) was then disrupted by treatment with Tyrode's solution containing 2 µM ryanodine (Abcam Biochemicals, UK) for up to 45 min. This concentration of ryanodine has been shown to deplete the sarcoplasmic reticulum Ca^{2+} stores and slow the spontaneous beating rates of sinus node myocytes.[3] After ryanodine treatment, the right atrial preparations were frozen and saved for structural and biochemical microscopic investigations.

Tissue freezing

After electrophysiological investigations, tissue was pinned onto a piece of cork. The cork was marked with a cut and a drawing was taken to note the orientation of the tissue. O.C.T. compound (VWR International, UK) was applied in between the cork piece and tissue, and over the tissue before rapidly freezing the tissue by pouring on 2-methylbutane (Sigma-Aldrich, UK) cooled in liquid nitrogen. Separately, 10 whole hearts (4 *Serca2* FF and 6 *Serca2* KO) were quickly removed and frozen in liquid nitrogen cooled 2-methylbutane. Frozen samples were stored at -80°C until further use.

Tissue cryosectioning

Frozen right atrial preparations and whole hearts from *Serca2* FF and *Serca2* KO mice were sectioned using a Leica CM3050 S cryostat (Leica Microsystems, Germany). The cryostat chamber and tissue samples were maintained at -17°C and -18°C respectively, during sectioning. Frozen tissue samples were affixed to a 25 mm specimen disc (Leica Microsystems, Germany) with O.C.T. compound and cryosectioned. Right atrial tissue preparations were serially cut perpendicular to the crista terminalis from the superior vena cava to the inferior vena cava and used for characterising atrial and sinus node tissue. Serial cross sections of ventricular myocardium were cut from the apex to the base of the heart in a parallel manner and used for characterising the ventricular myocardium. Sections were cut at 20 µm thickness for immunolabelling and at 10 µm thickness for histology. The serial tissue sections were placed onto SuperFrost Plus slides (VWR International, UK) and stored at -80°C until use.

Western blot

Protein was isolated from the left and right ventricular free walls and the ventricular septum of 4 wild type and 6 knockout mice by homogenizing frozen tissue in RIPA buffer: 10 ml PBS, 10 ml 10% IGEPAL CA-630 (Sigma Aldrich), 5 ml 10% Na Deoxycholate (Sigma Aldrich), 1 ml 10% SDS (BDH), 0.5 ml 100 mM PMSF (Sigma Aldrich), 50 µl 1mg/ml leupeptin (Sigma Aldrich), 50 µl 2 mg/ml aprotinin (Sigma Aldrich), 0.25 µl 1 mg/ml pepstatin (Sigma Aldrich), 73.15 ml deionized water. Homogenate was then centrifuged at 3000 rpm for 5 min at 4 ºC and the supernatant collected. Protein yield was quantified using Qubit protein assay (Life Technologies). 50 µg protein was used for each sample and volume made up to 7.5 µl with deionized water. 2.5 µl NuPAGE LDS sample buffer was added to each sample. Samples were loaded onto NuPAGE 4-12% Bis-Tris gels (Life Technologies). NuPAGE MOPS SDS running buffer (Life Technologies)

was used and electrophoresis run at 200 V constant for 50 min. Western blot was performed using a Trans-Blot Turbo Transfer System (Bio-Rad) and Trans-Blot Turbo Mini Nitrocellulose Transfer Packs (Bio-Rad). Transfer was performed using the Turbo protocol (25 V for 7 min). Transfer was checked by staining membranes with Ponceau red solution for 5 min followed by rinsing in deionized water. After staining, membranes were cleared by washing for 30 s in 0.1 M NaOH solution. Membranes were blocked overnight at 4 ºC in 3% BSA (Sigma Aldrich) in TBS-T: 100 ml TBS 10x (24.23 g Trizma HCl, 80.06 g NaCl in 1 l deionized water, pH 7.6), 900 ml deionized water, 1 ml Tween20 (Sigma Aldrich)). Membranes were washed 3 times in TBS-T followed by staining with rabbit polyclonal anti-SERCA2a IgG (Badrilla) and mouse monoclonal HRP-conjugated anti-α Tubulin IgG (Abcam) primary antibodies diluted in 1% TBS-T to a concentration of 1:2000 for 2 h at room temperature. Membranes were washed 3 times in TBS-T. Anti-rabbit HRP-conjugated secondary antibody (Cell Signalling Technology) diluted to 1:2000 in 1% BSA in TBS-T was applied to membranes for 2 h at room temperature. Membranes were washed 3 times in TBS-T. Amersham ECL detection reagents (GE Healthcare) were used to visualise signal using the Bio-Rad ChemiDoc MP imaging system (Bio-Rad). Band density was quantified using Image Studio Lite (LI-COR), and normalised to the expression of α-tubulin to correct for variation in protein loading.

Histology (Masson's trichrome staining)

Histological staining on atrial tissue sections cut perpendicular to the *crista terminalis* was carried out as described previously by Dobrzynski et al.[4-6] Briefly, Masson's trichrome stain was conducted on every 10th right atrial and ventricular tissue sections to determine the location, morphology and architecture of the atrial muscle and sinus node. Stained tissue sections were mounted in DPX mountant (Sigma-Aldrich, UK) and visualised using a Zeiss SteREO Discovery V8 microscope and histology images were captured via a Zeiss Axiocam MRc camera using Axiovision software (Carl Zeiss, Germany). Masson's trichrome stains nuclei dark blue/black, connective tissue blue and the cardiomyocytes pink/purple.

Immunohistochemistry protocol

Frozen tissue sections were fixed in 10% formalin in phosphate buffered saline (PBS; Sigma-Aldrich, UK) for 30 min in a fume cupboard, washed with PBS (Sigma-Aldrich, UK) three times (10 min each wash). The plasma membrane of fixed tissues were permeabilised by treating with PBS containing 0.1% Triton X-100 (Sigma-Aldrich, UK) for 30 min. Tissues were then washed three times in PBS (10 min each wash) and blocked using 1% Bovine Serum Albumin (BSA) (Sigma-Aldrich, UK) solution that was made in PBS (30 min blocking). Following a further three washes with PBS (10 min each), tissue sections were incubated with the appropriate primary antibodies overnight at 4°C. Primary antibodies were diluted in 1% BSA in PBS or 1% BSA 0.1% Triton X-100 and 10% mouse serum (Sigma-Aldrich, UK) in PBS in experiments where optimisation of the protocol and antibodies was being tested or when mouse raised primary antibodies were used upon mouse tissue. Antibody dilutions tested for optimisation included 1:100, 1:300, 1:500, 1:1000 and 1:3000 alongside a negative control (i.e. no primary antibody, but application of the secondary antibody on the tissue section, Supplement Figure 1). The following day, tissue sections were washed 3 times in PBS (10 min each) and incubated for 1 h with the secondary antibody at dilutions of 1:400 for Cy3-conjugates and 1:100 for FITC-conjugates diluted in 1% BSA in PBS. The primary and secondary antibodies used in this study are listed in Supplement Tables 1 and 2, respectively.

Confocal microscopy and image analysis

Immunofluorescence images of tissue sections were captured via a laser scanning confocal microscope (model LSM5), on the Axiocam HRc camera (Carl Zeiss, Germany). LSM5 Zeiss Pascal software was used for image acquisition. An appropriate laser, filters (FITC/Cy3) and wavelength of light were used to detect the fluorophores. One section from each heart was used for each antibody. Four images were obtained from each tissue section. Fluorescent images were quantified using Volocity software version 4.2.2 (PerkinElmer, U.K.). The signal intensity from each image (atrial, sinus node and ventricular tissue section images) was quantified and the values were averaged. For the atrial muscle and sinus node tissue images 'voxel spy' was used on Volocity software to reduce non-specific background signal. The 'find objects by intensity (range 20 – 255)' and 'exclude objects by size (<150)' were selected.

Supplementary Methods: AV node function in rats

Tissue preparation for electrophysiology

Male, Wistar-Hanover rats (n=13; 3 month old; Charles River, UK) were used to study the role of sarcoplasmic reticulum Ca^{2+} in AV node conduction. Rats were sacrificed by stunning and cervical dislocation and the heart was quickly excised and dissected in Tyrode's solution. Tissue preparation with intact sinus node and AV node was transferred to the recording chamber perfused with Tyrode's solution at 37±0.5°C at a rate of 20-25 ml/min. Extracellular electrograms were recorded using four bipolar electrodes made using 0.25 mm silver wire with 0.04 mm Teflon coating (Advent Research Materials, Oxford) and ~0.2 mm inter-pole distance. Signals were amplified 5000 times (NL104A, Digitimer, UK), filtered between 50 – 500 Hz (NL125/6, Digitimer, UK) and digitised (Micro 1401, Cambridge Electronic Design, UK) for storage on a computer. Spike 2 software (Cambridge Electronic Design, UK) was used for data acquisition and analyses.

In vitro electrophysiology

Tissue preparations were allowed to equilibrate for 20 min prior to electrophysiological recordings. One electrode was positioned at the site of earliest activation (sinus node), the second electrode was positioned on the atrial septum between the fossa ovalis and the tricuspid valve annulus in a location along the 'fast pathway' into the AV node. A third electrode was placed between the coronary sinus and the tricuspid valve annulus along the 'slow pathway' into the AV node. The fourth bipolar electrode was placed near the third electrode and moved along the tricuspid valve annulus using the micromanipulator until both an atrial and a second discrete sharp His signal was recorded.

The spontaneous sinus node cycle length was measured. Tissue was overdrive paced at the level of the sinus node by a continuous train of square pulse of 2 ms duration and twice the threshold voltage (minimum voltage required to capture the atrium) at an interval of 180 ms. The time between the atrial and His signal on the electrogram (4th electrode) was measured as the AH interval. Wenckebach cycle length was determined using a S1-S2 protocol. Tissue was paced at 200 ms intervals for 30 s and if 1:1 atrial to ventricular conduction occurred, the S1-S2 protocol was repeated with 10 ms reductions in cycle length until Wenckebach conduction was observed. At this point, an additional set of 30 s S1-S2 protocols was undertaken starting at 11 ms longer than the cycle length at which Wenckebach conduction had occurred. If 1:1 atrial to ventricular conduction occurred, the S1-S2 protocol was repeated with 1 ms reductions in cycle length. The Wenckebach cycle length was recorded as the cycle length at which 1:1 atrial to ventricular conduction failed and

Wenckebach conduction occurred. The spontaneous sinus node cycle length, the AH interval and Wenckebach cycle length measurements were repeated in the presence of ryanodine (2 µM).

Supplementary Methods: Computational modelling of mouse heart rate

The aim of the computational modelling was to dissect the effects of alteration of pacemaker components on the sinus node action potential to uncover mechanisms determining the heart rate in *Serca2* KO mice. The effect of the parameter alterations on the heart rate, in beats per minute, was recorded. First, we performed a simulation to elicit the relationship between SERCA2 downregulation (0-99%) and heart rate. This simulation formed the baseline for subsequent simulations. Secondly, the baseline simulation was repeated by altering the function of the sarcoplasmic reticulum Ca^{2+} release channel, the ryanodine receptor (RyR2). A third simulation was performed with an augmented level of the Cav1.3 L-type Ca^{2+} current ($I_{Ca,L}$). The fourth simulation was performed with an augmented Na^{+}-Ca^{2+} exchanger current (I_{NaCa}) conductance. The augmentation of $I_{Ca,L}$ and I_{NaCa} was implemented as 1.3 and 2 fold increase in the maximum conductance respectively. This is in accordance to experimental data obtained in isolated ventricular myocytes of *Serca2* KO mice with ~99% SERCA2a downregulation.[7] Finally, all of the above alterations were implemented simultaneously to permit examination of the overall remodelling on heart rate in *Serca2* KO mice.

Limitations of modelling:

The experimental data regarding phosphorylation may be relevant, especially in the case of regulating SERCA2. However, the effects of phosphorylation can be considered to be incorporated in the conductance alterations. With regards to the electrophysiological parameters affecting heart rate, we have not investigated the effects of the biophysical parameters, i.e. gating parameters of channels, instead only maximum conductance were altered for the sake of simplicity. The experimental data in this study were obtained from tissue preparations and the whole animal, the modelling is based on single cell simulations. This is justified since the sinus node cells initiate the propagation. Therefore, we focused on sinus node cell electrophysiology. In future studies that will extend this experimental-modelling work by incorporating new experimental data (e.g. any K^{+} channel conductance changes), in depth tissue simulations can be performed using this model.

Supplementary Tables

Supplement Table 1: Primary antibodies.

Primary antibody	Manufacturer	Animal antibody raised in	Monoclonal/ polyclonal	Antibody isotype/ dilution
SERCA2a anti-serum	Badrilla (Cat#: A010-20)	Rabbit	Polyclonal (SERCA2a specific)	IgG/1:500
Anti-connexin -43 antibody	Sigma Aldrich (Product#: C6219)	Rabbit	Polyclonal	IgG/1:1000
Anti-HCN4	Alomone Labs (Cat#: APC-052)	Rabbit	Polyclonal	IgG/1:100

Supplement Table 2: Secondary antibodies.

Secondary antibody	Manufacturer	Specificity	Wavelength (nm)	
			Absorption peak	Emission peak
Donkey anti-mouse IgG affinity purified; Cy3 conjugated	Millipore (Cat#: AP192C)	Mouse IgG	550	570
Donkey anti-rabbit (H+L) Cy3 conjugated affinity purified	Millipore (Cat#: AP182C)	Rabbit IgG	550	570
Donkey anti-rabbit IgG affinity Purified, fluorescein conjugated absorbed for dual labelling secondary antibody	Millipore (Cat#: AP182F)	Rabbit IgG	492	520

Supplementary Figures and Figure Legends

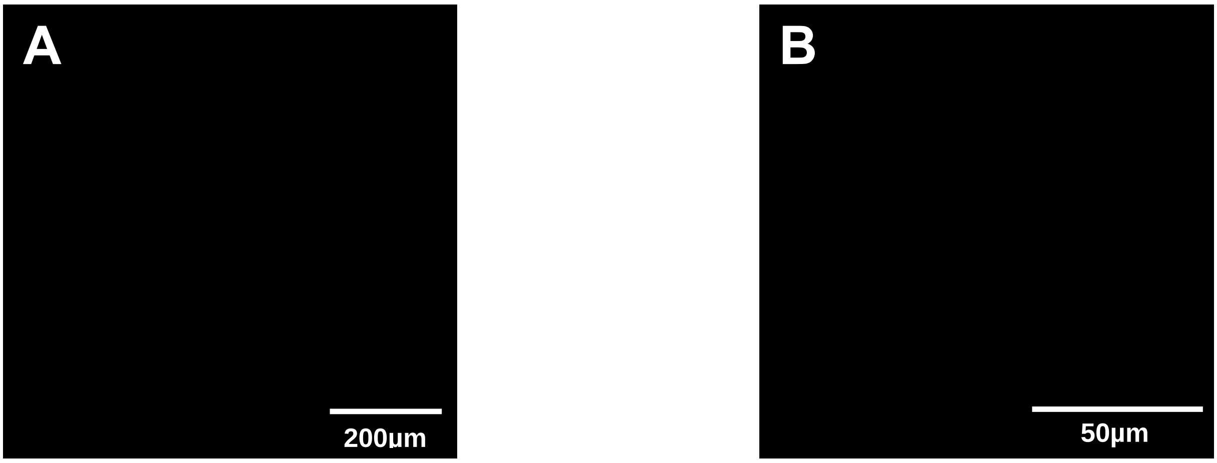

Supplement Figure 1. Immunofluorescence negative controls. Low (A) and high (B) magnification images of ventricular tissue cross section from a *Serca2* KO heart. Tissue section was treated identical to that shown in Figure 3D, with the exception of primary antibody.

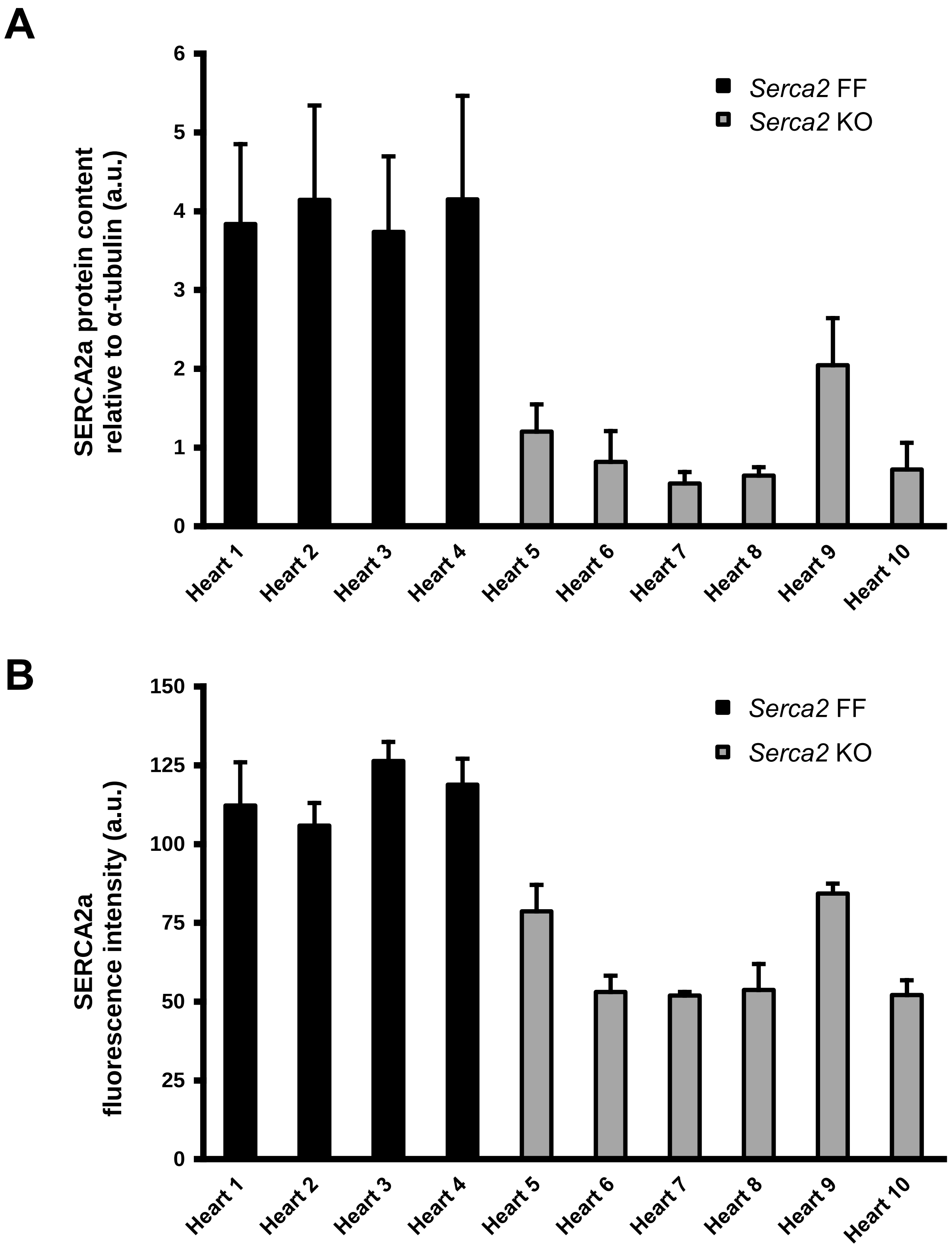

Supplement Figure 2. Quantification of SERCA2a protein expression in ventricular myocardium. A, Western blot SERCA2a protein band density normalised to α-tubulin in *Serca2* FF (black bars) and *Serca2* KO (grey bars) hearts. Mean data of three tissue samples (left ventricle, right ventricle and ventricular septum) collected from each heart is shown. B, immunofluorescence intensities of SERCA2a protein labelling in ventricular myocardium of *Serca2* FF (black bars) and *Serca2* KO (grey bars) hearts. Fluorescence intensity measurements shown are from four different regions of each heart section. Data are presented as mean ± s.e.m. (*Serca2* FF, n=4 and *Serca2* KO, n=6)

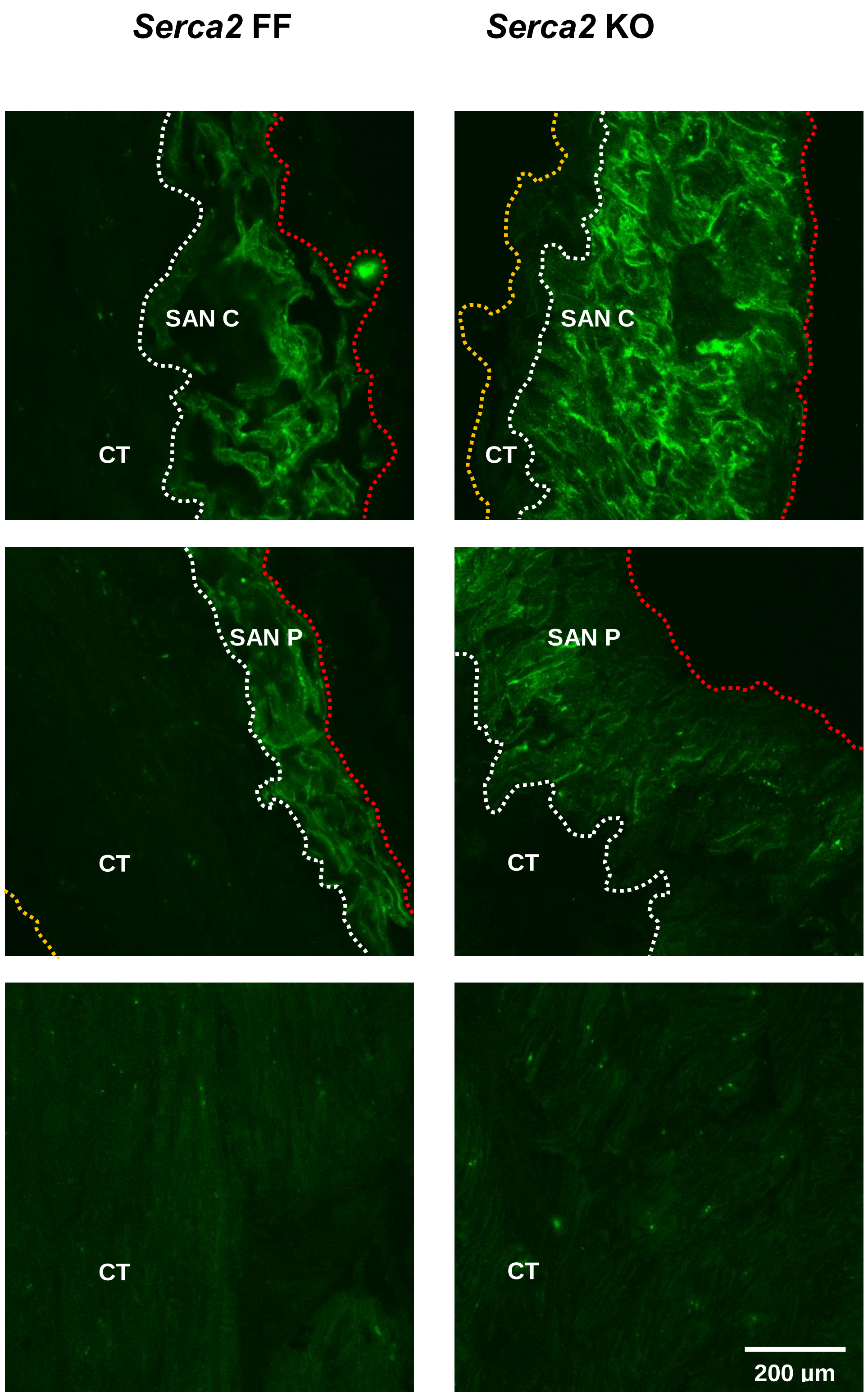

Supplement Figure 3. HCN4 expression in the right atrium. A, representative high magnification images showing HCN4 labelling in the sinus node centre (SAN C; top row), sinus node periphery (SAN P; middle row) and the atrial muscle of the crista terminalis (CT; bottom row) of *Serca2* FF (left images) and *Serca2* KO (right images) right atrial tissue sections. Red and yellow dotted lines represent epicardium and endocardium respectively and white dotted lines indicate the boundary between the sinus node and atrial muscle.

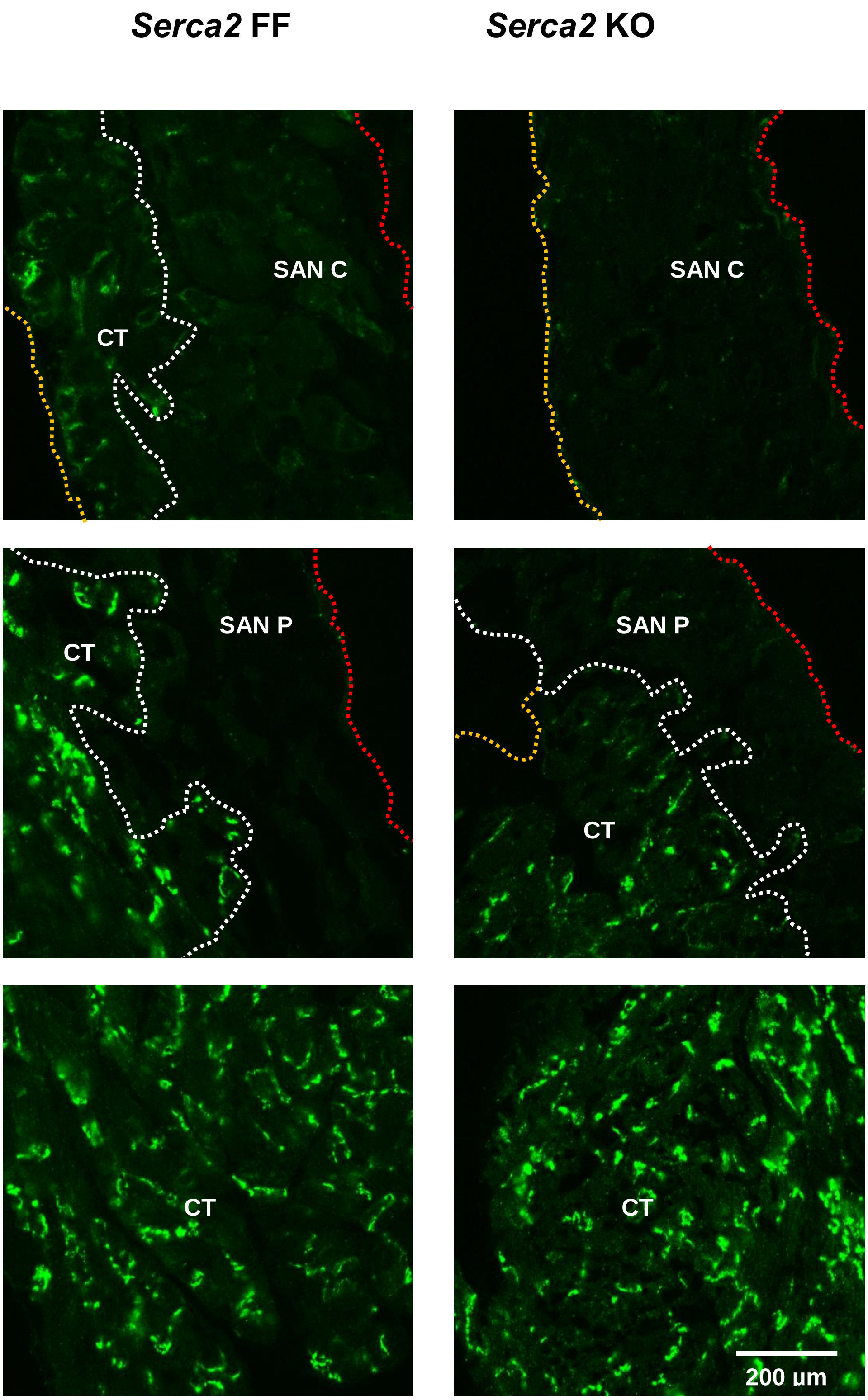

Supplement Figure 4. Cx43 expression in the right atrium. A, representative high magnification images of Cx43 labelling (green signal) in sinus node centre (SAN C; top row), sinus node periphery (SAN P; middle row) and atrial muscle of the *crista terminalis* (CT; bottom row), of *Serca2* FF (left images) and *Serca2* KO (right images) right atrial tissue sections. Red and yellow dotted lines represent epicardium and endocardial surface respectively and white dotted lines indicate the boundary between the sinus node and atrial muscle.

References

1. Stokke MK, Hougen K, Sjaastad I, Louch WE, Briston SJ, Enger UH, et al. Reduced SERCA2 abundance decreases the propensity for Ca^{2+} wave development in ventricular myocytes. Cardiovasc Res. 2010; 86:63-71.
2. D'Souza A, Bucchi A, Johnsen AB, Logantha SJ, Monfredi O, Yanni J, et al. Exercise training reduces resting heart rate via downregulation of the funny channel HCN4. Nat Commun. 2014; 5:3775.
3. Lancaster MK, Jones SA, Harrison SM, Boyett MR. Intracellular Ca^{2+} and pacemaking within the rabbit sinoatrial node: heterogeneity of role and control. J Physiol. 2004; 556:481-494.
4. Dobrzynski H, Billeter R, Greener ID, Tellez JO, Chandler NJ, Flagg TP, et al. Expression of Kir2.1 and Kir6.2 transgenes under the control of the alpha-MHC promoter in the sinoatrial and atrioventricular nodes in transgenic mice. J Mol Cell Cardiol. 2006; 41:855-867.
5. Dobrzynski H, Nikoloski VP, Sambelashvili AT, Greener ID, Boyett MR, Efimov IR. Site of origin and molecular substrate of atrioventricular junctional rhythm in the rabbit heart. Circ. Res. 2003; 93:1102-1110.
6. Dobrzynski H, Li J, Tellez J, Greener ID, Nikolski VP, Wright SE, et al. Computer three-dimensional reconstruction of the sinoatrial node. Circulation. 2005; 111:846-854.
7. Andersson KB, Birkeland JA, Finsen AV, Louch WE, Sjaastad I, Wang Y, et al. Moderate heart dysfunction in mice with inducible cardiomyocyte-specific excision of the *Serca2* gene. J Mol Cell Cardiol 2009; 47:180-187.
